# Fuzzy Logic in Aircraft Onboard Systems Reliability Evaluation—A New Approach

**DOI:** 10.3390/s21237913

**Published:** 2021-11-27

**Authors:** Andrzej Żyluk, Konrad Kuźma, Norbert Grzesik, Mariusz Zieja, Justyna Tomaszewska

**Affiliations:** 1 Air Force Institute of Technology, Księcia Boleslawa 6, 01-494 Warsaw, Poland; andrzej.zyluk@itwl.pl (A.Ż.); mariusz.zieja@itwl.pl (M.Z.); 2 Faculty of Aviation, Military University of Aviation, Dywizjonu 303 No. 35, 08-521 Deblin, Poland; k.kuzma@law.mil.pl (K.K.); n.grzesik@law.mil.pl (N.G.)

**Keywords:** aircraft airborne systems, air armament, reliability assessment, fuzzy logic, expert systems, Matlab

## Abstract

This paper is a continuation of research into the possibility of using fuzzy logic to assess the reliability of a selected airborne system. The research objectives include an analysis of statistical data, a reliability analysis in the classical approach, a reliability analysis in the fuzzy set theory approach, and a comparison of the obtained results. The system selected for the investigation was the aircraft gun system. In the first step, after analysing the statistical (operational) data, reliability was assessed using a classical probabilistic model in which, on the basis of the Weibull distribution fitted to the operational data, the basic reliability characteristics were determined, including the reliability function for the selected aircraft system. The second reliability analysis, in a fuzzy set theory approach, was conducted using a Mamdani Type Fuzzy Logic Controller developed in the Matlab software with the Fuzzy Logic Toolbox package. The controller was designed on the basis of expert knowledge obtained by a survey. Based on the input signals in the form of equipment operation time (number of flying hours), number of shots performed (shots), and the state of equipment corrosion (corrosion), the controller determines the reliability of air armament. The final step was to compare the results obtained from two methods: classical probabilistic model and fuzzy logic. The authors have proved that the reliability model using fuzzy logic can be used to assess the reliability of aircraft airborne systems.

## 1. Introduction

Taking into account the increasing complexity of technical objects and the fact that their components interact with each other, reliability is determined using experimental data obtained during exploitation or during planned reliability tests [1]. The methods of forecasting reliability play an important role in this respect. Forecasting is a prediction of future events, phenomena, or facts on the basis of premises determined by scientific research. Reliability forecasting is inseparably connected with conducting a proper diagnostic process, in which a diagnostic analysis of factors influencing damage should be carried out. It is also necessary to choose appropriate mathematical methods that are able to approximate the changes occurring in a given object in the near future or in a longer term. Numerous forecasting methods can be found in the available literature. A uniform classification of these methods is not widespread; however, some experts divide them into two main groups: quantitative (statistical–mathematical) methods and qualitative (non-mathematical) methods [2]. The quantitative methods are based on numerical data, as opposed to qualitative methods, which are based mainly on judgements or expert opinions.

In the case of technical facilities, the research conducted to date has shown the applicability of the following forecasting models for determining reliability:

(a) time series extrapolation values [3],
(b) adaptive trend models [4],
(c) autonomous extrapolation of stochastic processes [5],
(d) probabilistic models [6],
(e) expert methods [7,8].

In probabilistic models, reliability forecasting is based on knowledge of the probability distribution of the random variable T and the functional forms of the reliability indicators. These models are mainly used when functional reliability indicators can be determined on the basis of reliability examinations. A mathematical distribution is then fitted to the empirically determined probability distribution of the random variable so as to describe the collected empirical data.

An air armament system, as with any other technical system, should be characterised by high reliability so that air missions can be performed without any disruptions. The reliability of a technical system largely depends on a failure-free operation of its individual components. The occurrence of a system failure is a random variable. Increasingly, engineers are unable to accurately determine the factors causing the failure. In the operation of armament systems, human factors play a significant role, whose momentary indisposition or error can contribute to generating the malfunction. In case non-technical damage factors are involved, fuzzy logic can provide a useful tool. Fuzzy logic is mainly intended to take into account the uncertainty associated with such factors and therefore provides a better approximation of reality when modelling changes in machinery during its operation [9].

An analysis of literature data on the use of fuzzy logic for reliability assessment is extensively discussed by the authors [9]. A literature review over the past years is presented. It identifies three main streams using fuzzy number theory issues in reliability assessment:

- Failure risk analysis and assessment.
  Fuzzy logic is mainly used to estimate risk and determine the probability of damage. An example research article describing this trend is a study showing the effective use of fuzzy numbers as inputs and outputs in the isobutane cylinder rupture risk analysis based on Fault Tree and Event Tree methods [10]. Other studies based on real source data of two Italian industrial plants (a tyre manufacturing company and a chemical plant) indicate that fuzzy logic can be successfully applied to quantify the risk of accidents at work [11]. Fuzzy logic was also used in aviation [12,13]. It was used to assess the risk of a helicopter crash depending on two factors: intensity of operations and the probability of a crash [14].

- Human factors leading to damage.
  Fuzzy logic is used to model the uncertainty associated with human error factors. Research [15] has indicated the possibility of using fuzzy logic to define quality standards for operations, maintenance, and production activities, which can significantly reduce errors made by oil refinery personnel. Fuzzy logic and expert judgement have also been successfully used to determine the probability of human error among nuclear plant operators. The research results presented in [16] demonstrate the effectiveness of using fuzzy logic to determine the significance of risk of human error. Other studies which use fuzzy logic, taking into account human error and uncertainty in failure data, aim at assessing the imprecise failure probability of level crossing systems in Morocco [17]. A further example of the application of fuzzy logic in aviation is the study of the influence of human factors on damage. It has been successfully used to assess basic event failure rates for safety-critical avionics systems [18].

- Adequate planning of maintenance, and consequently, prevention of damage.
  Fuzzy logic has also been successfully used for proper planning of maintenance, and thus, to prevent damage. In article [19], fuzzy logic was successfully applied to model imprecise answers in a reliability-centered maintenance (RCM) diagram to answer questions on the causes, symptoms, and types of failure. Additionally, a maintenance-oriented milling machine reliability study using fuzzy logic and comparing it with the conventional method allowed a more accurate determination of the causes and consequences of failure [20]. Fuzzy logic was also used to determine specific maintenance tasks used to make reactions in chemical plants, based on equipment operating data. Along with neural networks, fuzzy logic complemented the RCM strategy [21].

In all of the analysed studies concerning the application of fuzzy logic for reliability assessment, as mentioned in the paper [22], the inaccuracy of the uncertainties in the reliability estimation is due to the fact that failures are rare events. Especially in aviation, very high priority is given to their elimination, often creating very expensive procedures as well as preventive facilities. This is what distinguishes aviation from other engineering sciences, where failure results in monetary costs, whilst in aviation, possible failure can cost human lives. Therefore, the authors decided to use fuzzy logic to identify and analyse rare cases such as the failure of aircraft airborne systems. This approach was motivated in the work [22], in which the authors by fuzzy logic mean fuzzy set theory and possibility theory. Taking into consideration such a definition, the authors aim at checking whether the use of fuzzy logic alone may be used to assess the reliability of a selected onboard aircraft system. For this purpose, an innovative reliability model of airborne air armament, based on expert knowledge, and a fuzzy controller was developed.

## 2. Research Methodology

The statistical data taken into account in probabilistic models of reliability assessment indicate failure, yet they do not take into consideration the reasons for its occurrence [23]. Failures occur for a variety of reasons and depend on a range of variables. Therefore, it is rather difficult to predict when a malfunction may occur. An alternative solution to classical reliability estimation methods, in this case, can be adopting a fuzzy logic approach. 

When seeking an answer to the posed question of whether a reliability model of a selected airborne armament system can be built by means of fuzzy logic, the authors designed and implemented a research algorithm, which includes the following steps:

- Analysing the available literature with a particular emphasis on the applications of fuzzy logic and the reliability issues of technical systems [9];
- Analysing available methods for estimating the systems reliability in a mathematical approach;
- Computing the reliability indicators of a selected airborne armament system, by means of a mathematical approach (based on statistical data from an IT-based aircraft reliability analysis system);
- Developing a reliability model of a selected airborne armament system by means of fuzzy logic;
- Assessing the reliability indicators of the aircraft armament system on the basis of the developed model, by means of fuzzy logic (using input signals from an IT system for the aircraft reliability analysis);
- Comparing the results obtained in the mathematical approach and the fuzzy set theory approach;
- Formulating and presenting the conclusions.

## 3. Object of Research

For the purpose of the research, the authors selected the armament subsystem of the TS-11 “Iskra” aircraft, which can be regarded as an icon of Polish aviation. It is a two-seat jet trainer and light combat aircraft. It is designed for basic pilot training, training in flights in adverse weather conditions, aerobatic flights, training on the training ground (shooting, bombing), and for air photographic reconnaissance. The selected tactical and technical data of the TS-11 “Iskra” aircraft are shown in Table 1.

The TS-11 “Iskra” aircraft is fitted with missiles and shooting and bombing armaments. The shooting armament includes a NS-23KM (or, alternatively, NR-23) 23 mm calibre cannon with an ammunition reserve of 80 rounds for the combat version or 40 rounds for the training version. An electrical control of the cannon, missile firing, and bomb dropping is steered via two buttons located in the front upper part of the aircraft control stick by the pilot from the front seat. The preparation of the electrical installation for the use of the cannon or missiles is executed by means of a three-position switch located on the armament plate in the cockpit of the front cabin, by switching it to the appropriate position: “CANNON”, “GUN”, or “MISSILE”. There are also corresponding buttons on the armament plate for electro-pneumatic reloading of the cannon.

The NS-23KM cannon is mounted on the right side of the aircraft, rigidly attached to the front grate, without cushioning, by two nodes: the front one, called the cannon bedding, and the rear one. An additional gun mount node was used to reduce the barrel vibrations. When firing, the gun bedding takes the recoil force and the rear node serves as a support for the gun. The selected tactical and technical data of the TS-11 “Iskra” aircraft are shown in Table 2.

The gun’s ammunition handling set consists of an ammunition box and entrance and exit chutes.

The cannon reloading system is an electro-pneumatic system consisting of the EK-48 valve, compressed-air reservoir, cables, and the electrical control system for the EK-48 valve. Aiming, when shooting, is made possible by the ASP-3NM-1 collimator automatic sight. 

## 4. Scientific Approach

The research was divided into two main parts. In the first stage of the research, reliability indicators were determined using a probabilistic reliability model. In the second phase, the same reliability indicators were determined by means of a reliability model using fuzzy logic.

### 4.1. Analysis of Statistical Data

The damage statistics were obtained from the computerised support system of air operations.

**Figure 1:**
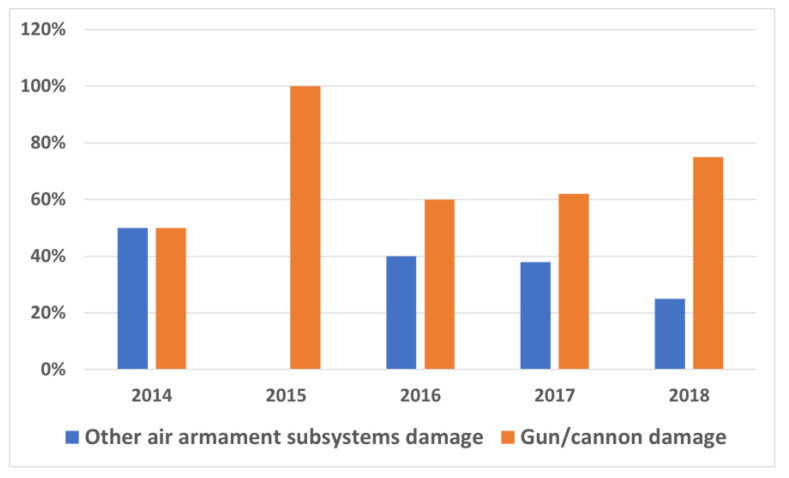
Percentage of gun/cannon damage in the total number of air armament damage from 2014 to 2018.

The sample batch was thirty TS-11 “Iskra” aircraft operated at a selected military air base. The analysis was conducted from 1 January 2014 to 30 November 2018. During this period, a total of 590 failures were recorded in all specialities, including 28 failures of aircraft weapons systems. Damage to air armaments accounts for approximately 5% of overall failures. When considering the guns and cannons, it needs to be noted that damage accounts for a significant portion of malfunctions of the entire armament. Figure 1 shows the percentage share of gun/cannon damage in relation to the other subsystems. In general, it accounts for approximately 67% of all damage to armament systems.

**Figure 2:**
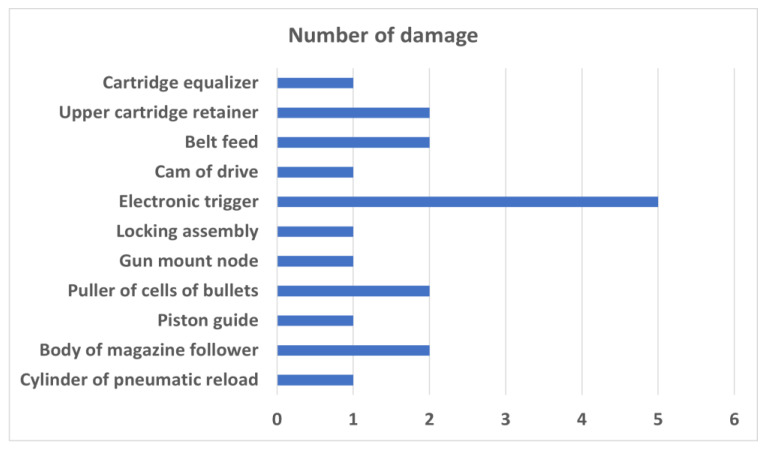
Distribution of damage of guns and cannons split into individual subsystems.

Among nineteen reported damages to the subsystem, the vast majority of malfunctions concerned the NS-23KM (interchangeably NR-23) cannon or the cannon’s mounting components. Eleven components were reported to be damaged. A numerical listing of the damage to individual components is shown in Figure 2.

The vast majority of the damage to these components, over 70%, was caused by material fatigue and excessive vibration during cannon firing. Based on the analysed data, it can be concluded that fatigue processes constitute a significant causal group of damage to the shooting armament of the TS-11 “Iskra” aircraft.

### 4.2. Probabilistic Model of Reliability of the Gun/Cannon Subsystem

In the developed probabilistic reliability model of the subsystem, the time to failure in the form of the number of flying hours was assumed as a non-negative random variable T. After initial data processing and a statistical analysis, the random variable was ranked from the smallest to the largest. Statistical observations were also assumed to be censored observations as the data for the study were taken from specific years and the study was completed on 30.11.2018. An important simplification in the model was to assume the gun/cannon subsystem as a non-repealable object. The initially compiled statistical data are shown in Table 3.

#### 4.2.1. Alignment of Measurement Results

The data from complete observations were checked for outliers. For this purpose, Grubbs’ criterion was used to reject questionable data [25]. The arithmetic mean of the random variable was calculated from formula:

(1) $$\bar{T}=\frac{1}{n}\overset{n}{\underset{i=1}{\sum }}T_{i}=\frac{4482}{19}=235.895$$

The standard deviation of the random variable was computed using the root of the unbiased estimator of variance:

(2) $$s=\sqrt{\frac{\sum_{i=1}^{n}{\left(T_{i}-\bar{T}\right)}^{2}}{n-1}}=\sqrt{\frac{13064.21053}{18}}=114.229$$

Next, the Grubbs’ coefficient was determined η from formula:

(3) $$\eta =\frac{\left|T_{i}-\bar{T}\right|}{s}$$

In Grubbs’ criterion, a measurement is rejected if there is a relationship:

(4) $$\eta_{q}\leq \;\eta$$

where:

$\eta_{q}$—Grubbs’ criterion.

Assuming a risk of error on the level of 5%, Grubbs’ criterion value of $\eta_{q}=2156$ was read out from the table. Since there was no relationship (4) for any measurement, none of the measurements were rejected. The calculated Grubbs’ coefficients for the 19 measurements of complete observations are shown in Table 4.

#### 4.2.2. Fitting the Distribution of a Random Variable

**Figure 3:**
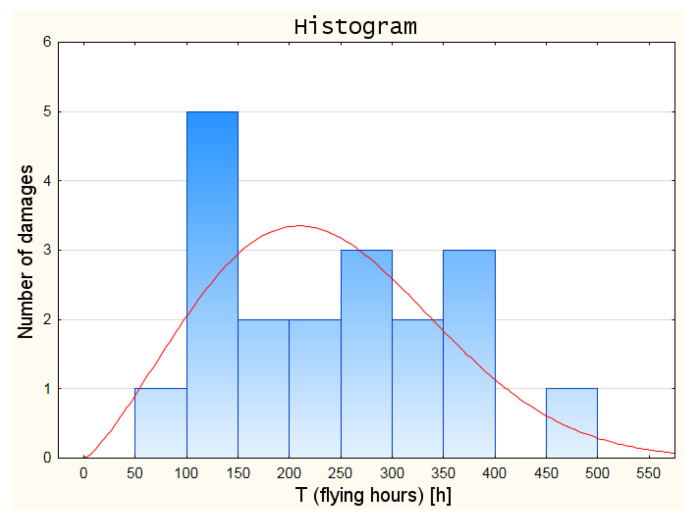
Histogram of gun/cannon damage.

On the basis of the ranked statistical data, which were further analysed, a histogram was created. This enabled a preliminary determination of which distribution of the random variable will be the most approximate one with regard to the empirical data. The damage histogram developed in Statistica software is shown in Figure 3. In addition, the Kolmogorov–Smirnov test was carried out in Statistica software to match the distribution of the random variable to the empirical data. The test results are shown in Table 5.

On the basis of the previously conducted statistical analysis of damage formation, as well as the observations of the histogram of the damage distribution series and the results of the Kolmogorov–Smirnov test, the Weibull distribution was adopted as the distribution of the random variable for further analyses.

The Weibull distribution is a parametric distribution, so the next step was to find its parameters. For this purpose, a graphical method for determining the parameters of the distribution of a random variable was used. The Weibull probability grid was developed in Statistica software, from which the scale parameter and the shape parameter of the distribution were read out. 

The shape and scale parameters estimated by the graphical method are as follows:

- shape parameter *α*—2.2664
- scale parameter *β*—318.78

Using the Weibull distribution, as well as its parameters estimated by the graphical method, functional reliability indicators were calculated for the empirical data:

- function of the intensity of the damage *λ*(*t*)
  (5) $$\lambda \;\left(t\right)=\frac{\alpha }{\beta^{\alpha }}t^{\alpha -1};\;\;\;\;\alpha ,\beta >0;\;t>0$$
- density distribution function *f*(*t*)
  (6) $$f\left(t\right)=\frac{\alpha }{\beta }{\left(\frac{t}{\beta }\right)}^{\alpha -1}e^{-{\left(\frac{t}{\beta }\right)}^{\alpha }}$$
- random variable distribution *F*(*t*)
  (7) $$Q\left(t\right)=F\left(t\right)=1-e^{-{\left(\frac{t}{\beta }\right)}^{\alpha }}$$
- the reliability function *R*(*t*).
  (8) $$R\left(t\right)=e^{-{\left(\frac{t}{\beta }\right)}^{\alpha }}$$

The graphic method of determining the Weibull parameters, due to its nature, may be subject to some error. Therefore, it was decided to analyse the data of the censored observations in Statistica software. For this purpose, the Process Analysis module, and in particular, Weibull Reliability/Damage Time Analysis, was used. In this analysis, the distribution parameters determined by the maximum reliability method differ only slightly from those determined by the graphical method. The resulting scale parameter was 0.7 smaller than the one that was estimated from the graphical method, while the shape parameter was approximately 0.3× larger. The values of the specific parameters are shown in Table 6.

The Weibull analysis in Statistica, both in the Kaplan–Meier estimate and Hollander–Proschan New-Better-Than-Used test, which compare the theoretical reliability function with the Kaplan–Meier estimate, showed that the proposed distribution describes data reasonably. 

Based on the parameters of the Weibull distribution obtained in the Weibull Reliability/Damage Time Analysis, it was possible to calculate the values of the intensity function, the distribution density function, the distribution of the random variable, and the reliability function for the individual times to failure.

The differences between the parameters of empirical Weibull distribution from the operational data and the graphical method of determining the parameters and theoretical Weibull distribution (Table 6) obtained as a result of Weibull Reliability/Damage Time Analysis in Statistica software result in slightly different values of the individual reliability functions. The values of the individual functions are within a 95% confidence interval, which means that with a probability of 95%, the reliability of the armament can be estimated from both empirical and theoretical distributions. In order to illustrate the differences between the values of the reliability function, the calculation results for the distribution density and the reliability function are shown in Table 7.

**Figure 4:**
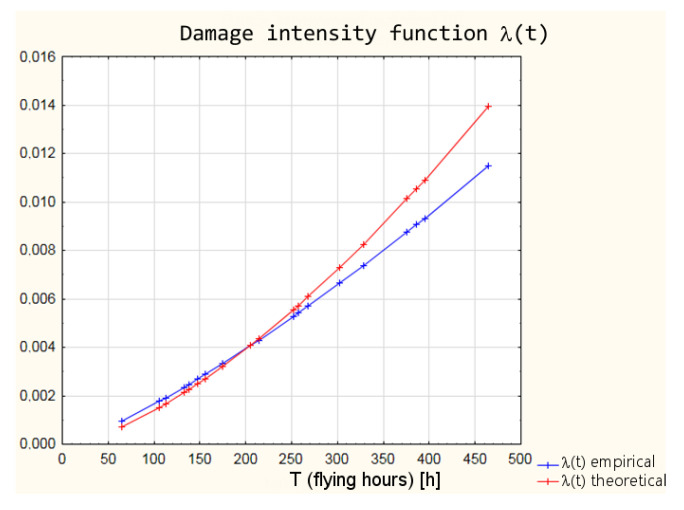
Comparison of empirical and theoretical damage intensity function of armament.

**Figure 5:**
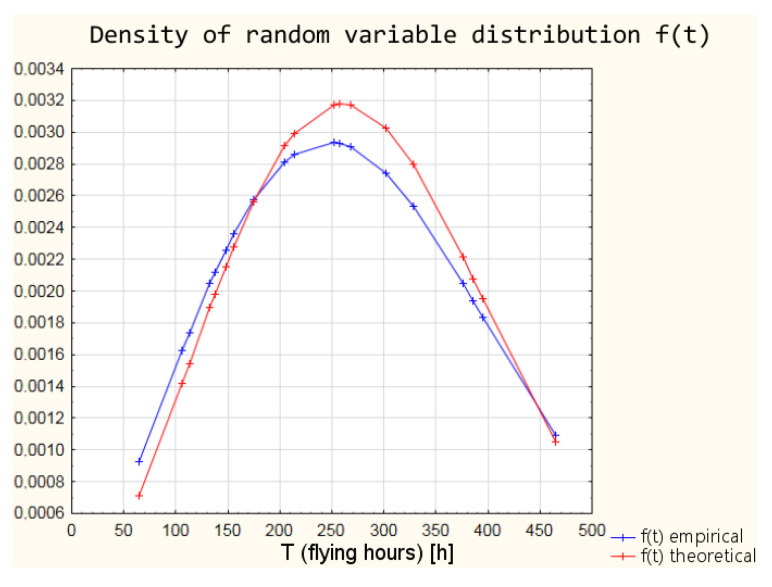
Comparison of empirical and theoretical density of random variable distributions.

**Figure 6:**
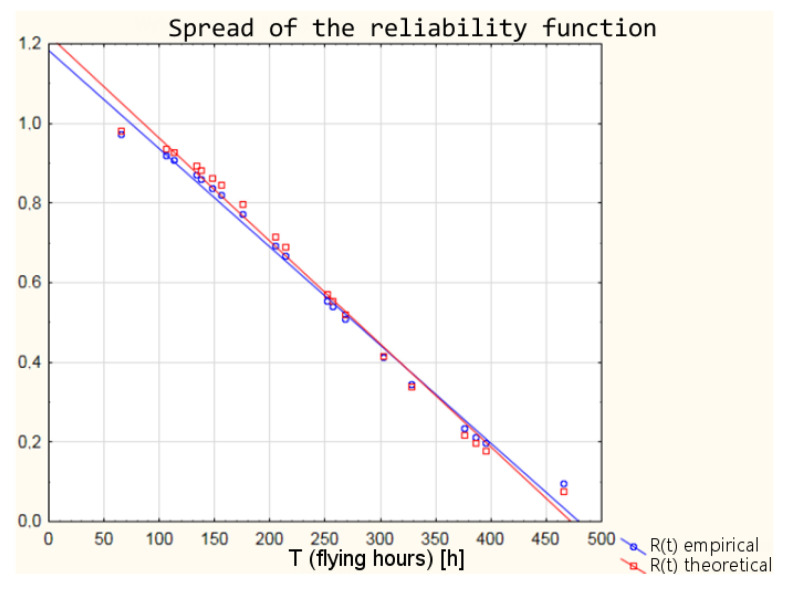
Discrepancy of empirical and theoretical values of the reliability function.

**Figure 7:**
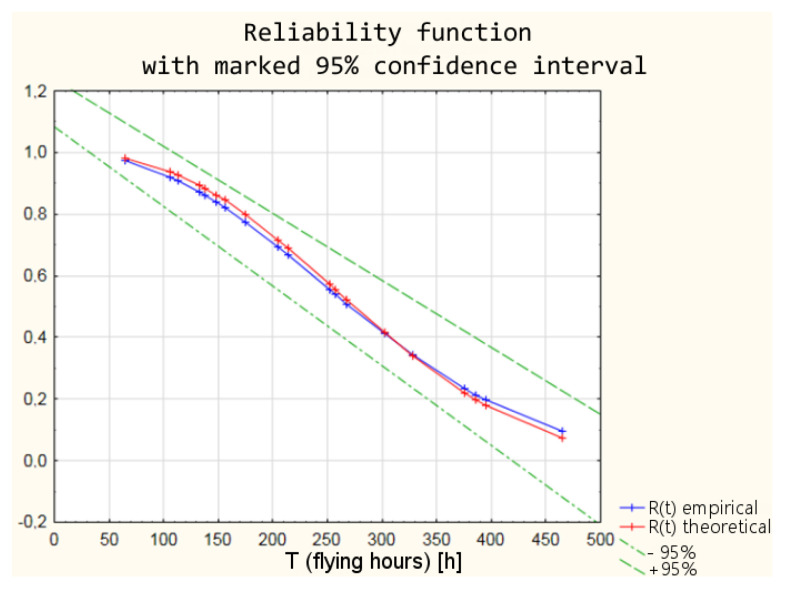
Comparison of empirical and theoretical reliability function with marked 95% confidence interval.

A graphic interpretation of the results of the two distributions is shown successively in Figure 4, Figure 5, Figure 6 and Figure 7.

### 4.3. Fuzzy Logic Reliability Model of a Firing Subsystem

The basis of the model using the fuzzy sets theory is a Mamdani fuzzy controller in MISO design. An extremely important issue in fuzzy modelling is the appropriate acquisition of expert knowledge. On this basis, the number and shape of membership functions of the input and output signals are determined, as well as the design of deduction rules base. With properly selected inference rules and the use of the right inference mechanism, the whole fuzzy modelling process takes place.

**Figure 8:**
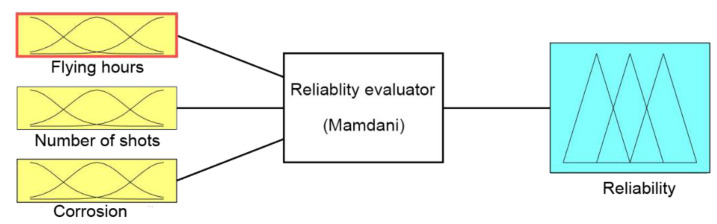
Diagram of the fuzzy controller.

In order to gain relevant expertise for the research, a questionnaire was developed and addressed to aviation weapons specialists who have at least 15 years experience on the equipment. Ten experts of the maintenance group dealing with repair and periodic maintenance of the aircraft armament TS-11 “Iskra” took part in the examination. Based on the information obtained in the survey, membership functions were determined, and a deduction rules base was built. A fuzzy controller was developed (Figure 8). 

In the designed model, the input signals are parameters that affect the reliability of armament flying hours, represented by the operating time of air assets, shots, represented by the operating time of armament and corrosion, which is characterised by the physical condition of the armament. For each input parameter, four triangular membership functions and one (last) trapezoidal one were adopted: “very small”, “small, “medium”, “large”, and “very large”, which define the range and characteristics of changes in the input parameters. For the output signal, which is used to determine the reliability of the armament, six triangular membership functions were adopted, respectively: “very small”, “small”, “medium”, “large”, “very large”, and “optimal”. The shape and range of the individual membership functions of the input and output signals are successively shown in Table 8, Table 9, Table 10 and Table 11.

The next step in designing the controller was to build the deduction principles base. At this stage of design, it was essential for the deduction principles to be complete. In [25], the number of principles computable for the same number of fuzzy sets of all model inputs was presented as:

(9) $$r_{w}=z_{r}{}^{w}$$

where:

$r_{w}$—number of deduction principles,

$z_{r}$—number of fuzzy sets of the model,

w—number of model inputs.

The number of fuzzy rules was calculated:

(10) $$r_{w}=5^{3}=125$$

The rules of deduction are expressed as, e.g.,

If the Number of Flying Hours is VERY SMALL and the Number of Shooting Hours is VERY SMALL and Corrosion is VERY SMALL, then the Reliability is OPTIMUM.

In the designed model, an inference mechanism based on Zadeh’s minimax method was chosen. The premises are connected by conjunction, so the conclusion is the minimum value of the activation membership functions. In the model, there are 125 principles involved in deduction. The evaluation of the premises is realised as a logical product (MIN operator), whereas the resulting membership function is realised as a logical sum (MAX operator), using an aggregation block.

The resulting membership function still belongs to the set of fuzzy numbers, so in order to obtain a single value at the output of the system, belonging to the set of real numbers, an appropriate defuzzification (sharpening) method should be applied. The centre of gravity method was used in the designed model. This method is one of the methods that takes into account all active rules in the defuzzification process. This guarantees the highest sensitivity to changes in the parameters of the input signals.

**Figure 9:**
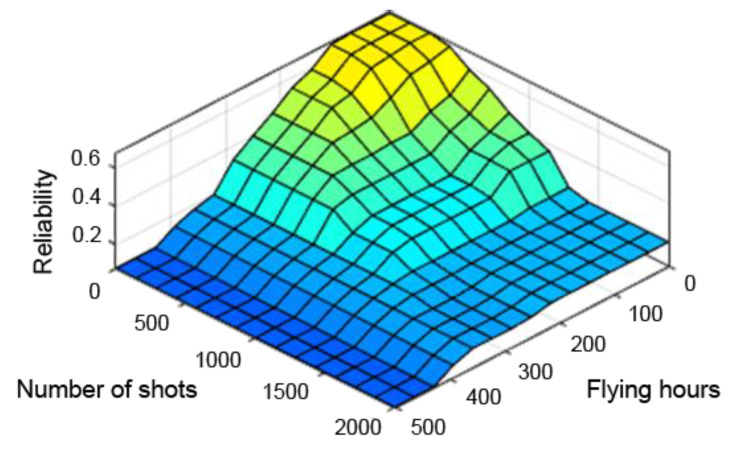
Reliability dependence on the number of flying hours and shooting hours.

**Figure 10:**
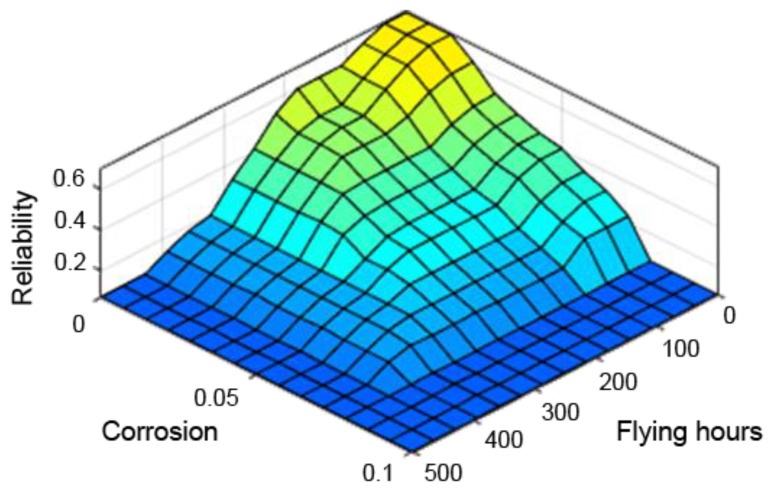
Dependence of reliability on the number of flying hours and corrosion.

**Figure 11:**
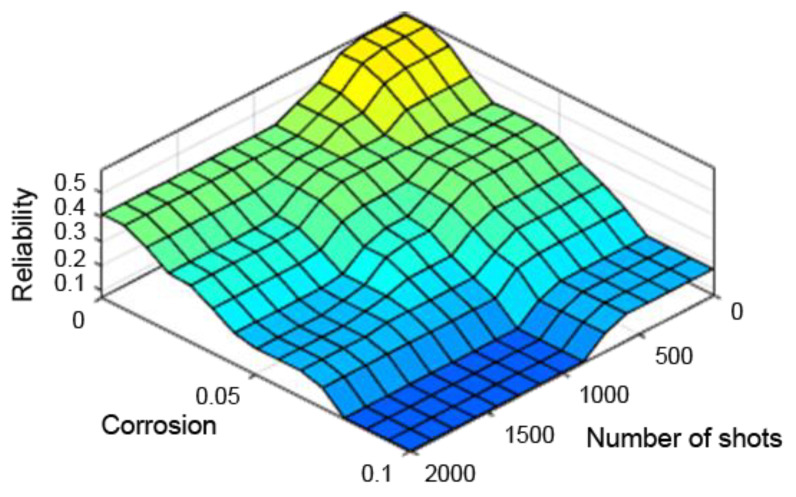
Dependence of reliability on the number of shooting hours and corrosion.

A very useful tool in assessing the performance of the designed fuzzy controller is the so-called “control planes (mapping planes)”, thanks to which it is possible to observe the nature of mapping “crisp” (not fuzzy) input values of the system into “crisp” values of the output variables over the entire range of parameters. The resulting control planes for the designed model are shown successively in Figure 9, Figure 10 and Figure 11.

Figure 9, Figure 10 and Figure 11 present control surfaces of the fuzzy logic controller. The surface on Figure 9 shows the influence of the number of firing rounds and flying hours on the gun reliability. We can observe on the chart that the reliability is the highest when the number of flying hours and rounds fired are the lowest. Figure 10 demonstrates influence of gun corrosion and flying hours on gun reliability. A similar situation as in Figure 9 can be observed, that reliability is the highest when corrosion and number of flying hours are the lowest. Figure 11 indicates the influence of gun corrosion and fired rounds on gun reliability. On that chart also is that the reliability is the highest when the corrosion and number of flying hours are the lowest. All the illustrations confirm that the designed fuzzy expert system works, and the received data are compatible to the technical manual of an aircraft gun.

Having analysed the control planes, it can be concluded that both the shape and boundaries of individual membership functions as well as their selection, and the deduction rules, based on expert knowledge, are appropriate. In order to validate the proper performance of the designed model, reliability model tests were performed.

### 4.4. Model Tests

The first stage of the model tests consisted of determining the reliability function of the armaments, in order to compare the results with the reliability function obtained from the mathematical model.

The discrete values of the flying hours were introduced into the model, using fuzzy logic, while assuming that the corrosion and shooting hours equal 0. This was to simulate the conditions under which the reliability was estimated in a classical probabilistic model. The resulting discrete values after undergoing the inference and defuzzification process are shown in Table 12.

**Figure 12:**
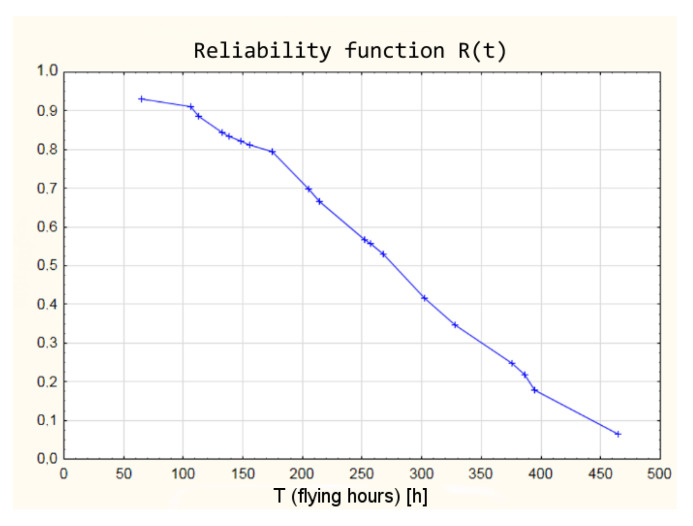
Reliability function derived from the fuzzy logic model.

A graphic interpretation, obtained from the fuzzy logic model, of the reliability function has been shown in Figure 12.

The next stage of the modelling research was to check how changing the defuzzification method affects the resulting reliability function. For this purpose, the method of the centre of the sums, the method of the centre of the maximum, and the first of the maximum method were checked successively.

**Figure 13:**
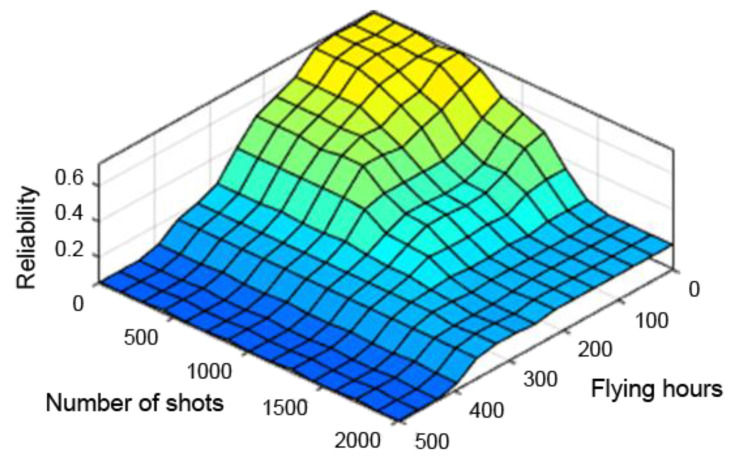
Reliability dependence on the number of flying hours and shooting hours with the centre of sums method.

**Figure 14:**
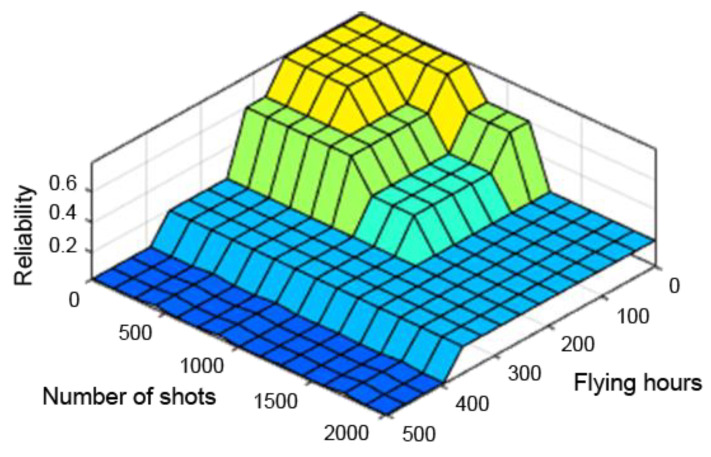
Reliability dependence on the number of flying hours and shooting hours with the middle of maximum method.

**Figure 15:**
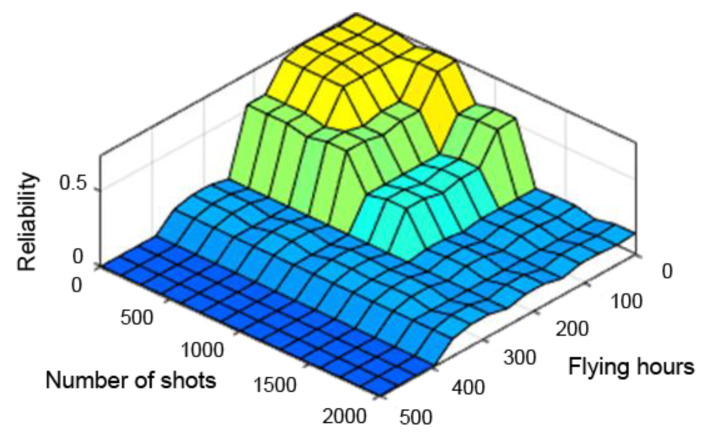
Reliability dependence on the number of flying hours and shooting hours with the first maximum method.

The observation of the control planes, obtained with the individual defuzzification methods already at an initial stage, excluded the first and middle maxima methods from further study. The control planes of these methods had too many abrupt changes, which would have improperly affected the results. Therefore, only the method of the centre of sums was adopted for further analysis. The control planes obtained from the different defuzzification methods are shown successively in Figure 13, Figure 14 and Figure 15.

The authors checked what values of the reliability function can be obtained by using the centre of sums method as a defuzzification mechanism. The obtained results are listed along with the results obtained with the centre of gravity as the defuzzification method, from Table 13.

**Figure 16:**
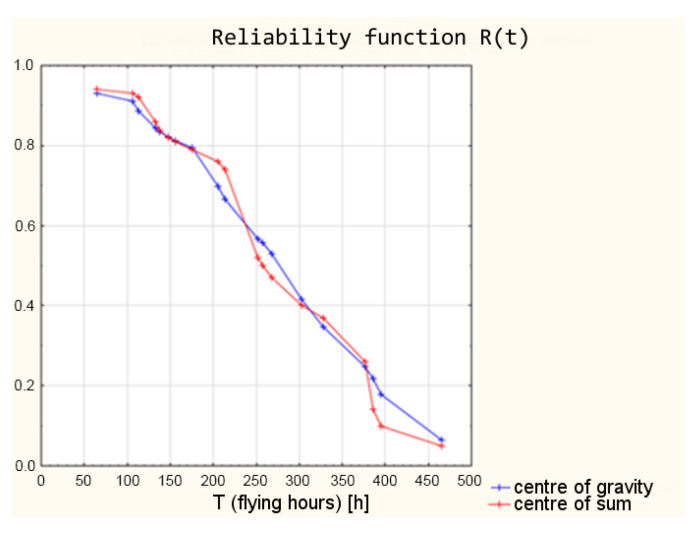
Reliability functions from a fuzzy logic model using the centre of gravity and the centre of sum defuzzification methods.

A graphical summary of the results using the centre of gravity and centre of sums is shown in Figure 16.

The analysis of the obtained values of the reliability function shows that, in this case, the centre of gravity is a better defuzzification method. The middle of the sums method gave results that were too abrupt given the shape of the reliability function from the mathematical model.

In addition, the authors tested whether changing the method of evaluating the premises as well as the method of aggregation in the resulting membership function affects the final results. Using different combinations of implication and aggregation methods did not produce significant differences in results.

### 4.5. Comparison of Research Findings

The classical probabilistic model of armament reliability developed on the basis of operational tests and the reliability indicators determined on its basis were the reference base to which the reliability results of the model using fuzzy logic were compared. Comparing the results should give a clear answer to the question posed by the authors of whether fuzzy logic offers the possibility of building a reliability model of the selected air weapon system. The results that were obtained from the reliability analysis in the classical approach and the fuzzy set theory approach have been listed in Table 14.

**Figure 17:**
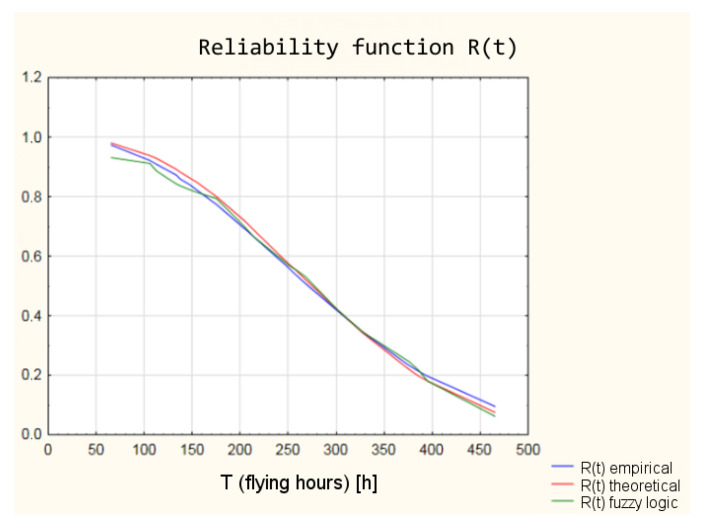
Shape of the reliability functions obtained from the classical and “fuzzy” model.

**Figure 18:**
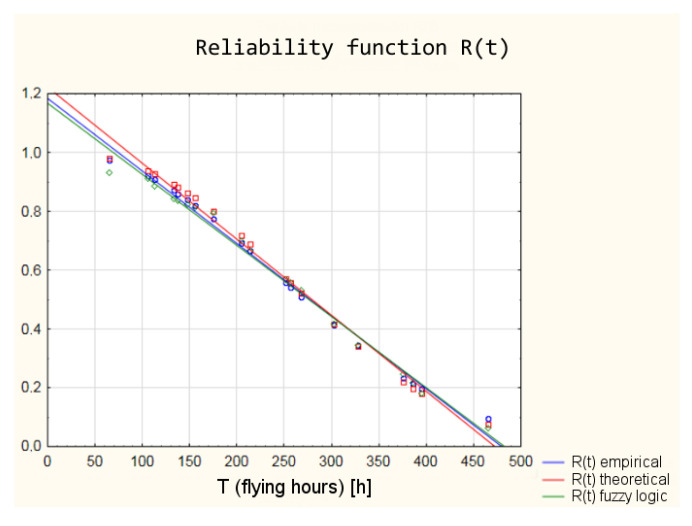
Discrepancy of the reliability function values obtained from the classical and the “fuzzy” model.

The graphic interpretation of the results is shown in Figure 17 and Figure 18.

**Figure 19:**
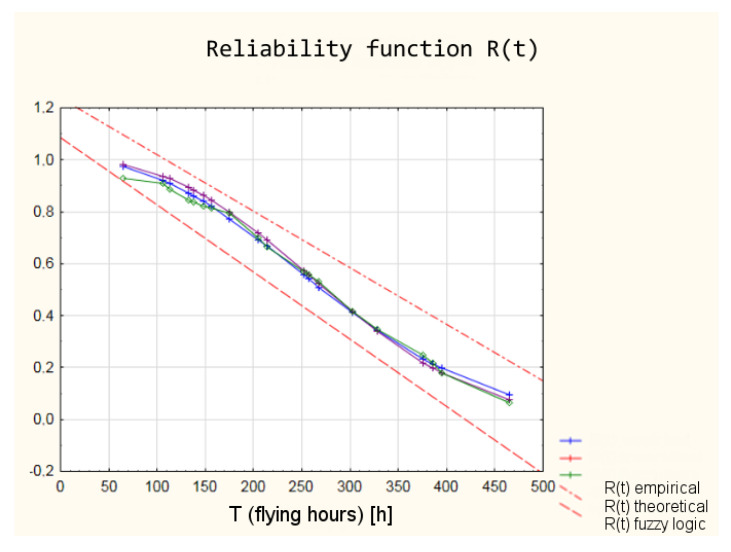
Reliability functions obtained from the classical and fuzzy model with marked 95% confidence interval.

The values of the reliability functions obtained from the model using fuzzy logic differ slightly from the values of the reliability functions determined using the empirical and theoretical Weibull distribution (analysis in the classical approach). These differences are within a 95% confidence interval (Figure 19), which means that with a probability of 0.95, the reliability of the armament of the TS-11 “Iskra” aircraft can be estimated by the developed model using fuzzy logic.

## 5. Conclusions

The execution of the assumed research objectives of the first part of the research included an analysis of statistical data and the reliability analysis of the selected air armament system in the classical approach. After analyzing the statistical (operational) data, the reliability was assessed using the classical probabilistic model, in which, on the basis of Weibull distribution fitted to the operational data, the basic reliability characteristics were determined, including the reliability function of the selected aircraft armament. This analysis has led to the following general conclusions:

- Fatigue processes are an important causal group of damage to the shooting armament of the TS-11 “Iskra” aircraft.
- The differences between the parameters of empirical Weibull distribution from the operational data and the graphic method of determining the parameters, and theoretical Weibull distribution are within the 95% confidence interval, which means that with a probability of 95%, reliability indicators from both the empirical and theoretical distribution can be used in further studies on the armament reliability.
- The interpretation and comparison of the results obtained from the reliability analysis in the classical approach and the reliability analysis in the fuzzy set theory approach allowed formulating the following conclusions:
- In order to develop a reliability model using fuzzy logic, access to reliable expert knowledge is necessary.
- Fuzzy logic offers a possibility to determine reliability based on various parameters and also allows an analysis and interpretation of the relationship between input parameter values and reliability.
- The developed reliability model using fuzzy logic can be used to assess the reliability of various systems without the need for knowledge of an extensive mathematical apparatus.
- The controller pattern, designed with a fuzzy logic reliability model, can be easily upgraded by changing the membership functions (shapes and limits) and the deduction principles.

The authors are aware that the subject matter covered in this article is a small section of a broader issue and further research is needed in this area. In particular, further research should include increasing the number of system input parameters affecting reliability to address possible errors in reliability estimation.

## Figures and Tables

**Table 1:** Basic tactical and technical data of the TS-11 “Iskra” aircraft [24].

Type	Data
Length	11.15 m
Wingspan	10.06 m
Height	3.50 m
Lifting surface	17.50 m²
Unloaded	2560 kg
Gross weight	3724 kg
Maximum take-off weight	3840 kg
Engine	WSK SO-3W with a thrust of 10.80 kN (1100 kG)
Maximum speed	720 km/h in 5000 m
Range	1260 km
Operational ceiling	11,000 m
Climb rate	14.8 m/s
Wing loading	213 kg/m²
Thrust-to-weight ratio	1:3.4

**Table 2:** Basic tactical and technical data of NS-23KM.

Type	Data
Calibre	23 mm
Rate of fire	500–590 rounds/min
Gun weight	37.5–38.2 kg
Length	1985 mm
Width	164 mm
Height	256 mm
Barrel length	1450 mm
Minimum air pressure required for reloading	30 kG/cm^2^

**Table 3:** Summary of sample performance data.

No	Observation	Flying Hours from 2014 to 2018	T (Flying Hours Until Malfunction [h])	Censored Observations
1	O10	313	65	complete
2	O9	80	80	censored
3	O14	95	95	censored
4	O5	249	106	complete
5	O8	391	113	complete
6	O3	148	133	complete
7	O11	305	138	complete
8	O1	296	148	complete
9	O28	310	156	complete
10	O24	247	175	complete
11	O7	204	204	censored
12	O21	410	205	complete
13	O4	246	214	complete
14	O12	232	232	censored
15	O22	243	243	censored
16	O26	251	251	censored
17	O29	423	252	complete
18	O23	314	257	complete
19	O2	278	268	complete
20	O25	278	278	censored
21	O30	294	294	censored
22	O27	297	297	censored
23	O16	453	302	complete
24	O6	307	307	censored
25	O13	310	310	censored
26	O15	442	328	complete
27	O20	490	376	complete
28	O18	448	386	complete
29	O19	477	395	complete
30	O17	519	465	complete

**Table 4:** Calculated Grubbs’ coefficients for complete observations.

No	Observation	Flying Hours from 2014 to 2018	Flying Hours Until Malfunction (h)	*η*
1	O10	313	65	1.495158054
2	O5	249	106	1.136449054
3	O8	391	113	1.075206054
4	O3	148	133	0.900226054
5	O11	305	138	0.856481054
6	O1	296	148	0.768991054
7	O28	310	156	0.698999054
8	O24	247	175	0.532768054
9	O21	410	205	0.270298054
10	O4	246	214	0.191557054
11	O29	423	252	0.140904946
12	O23	314	257	0.184649946
13	O2	278	268	0.280888946
14	O16	453	302	0.578354946
15	O15	442	328	0.805828946
16	O20	490	376	1.225780946
17	O18	448	386	1.313270946
18	O19	477	395	1.392011946
19	O17	519	465	2.004441946

**Table 5:** Matching the distribution of a random variable by means of the Kolmogorov–Smirnov test.

	d K-S	K-S p
Weibull	0.080785	0.980681
Generalised extreme value	0.080995	0.980185
Normal	0.083536	0.973478
Gaussian mixture	0.083917	0.972358
Johnson SB	0.086214	0.964943
Rayleigh	0.136471	0.584155
Log-normal	0.141784	0.536058
Triangular	0.161554	0.373656
Generalised Pareto	0.166667	0.337101
Semi-normal	0.240961	0.051135

**Table 6:** Differences in scale and shape parameters for empirical and theoretical Weibull distributions.

	Scale (*β*)	Shape (*α*)
Empirical Weibull distribution	318.78	2.2664
Theoretical Weibull distribution	318.06	2.5030

**Table 7:** Values of the distribution density *f*(*t*) and reliability function *R*(*t*) for empirical and theoretical Weibull distribution.

Flying Hours Until Malfunction (h)	Empirical Density of Distribution *f*(*t*)	Theoretical Density of Distribution *f*(*t*)	Empirical Reliability *R*(*t*)	Theoretical Reliability *R*(*t*)
65	0.00092	0.00071	0.97315	0.98139
106	0.00162	0.00142	0.92085	0.93809
113	0.00174	0.00154	0.90908	0.92774
133	0.00205	0.00190	0.87118	0.89335
138	0.00212	0.00198	0.86076	0.88365
148	0.00226	0.00215	0.83887	0.86298
156	0.00236	0.00228	0.82040	0.84526
175	0.00257	0.00256	0.77347	0.79919
205	0.00281	0.00292	0.69235	0.71672
214	0.00286	0.00299	0.66680	0.69012
252	0.00294	0.00317	0.55601	0.57214
257	0.00293	0.00318	0.54134	0.55626
268	0.00291	0.00317	0.50923	0.52132
302	0.00274	0.00303	0.41286	0.41546
328	0.00254	0.00280	0.34412	0.33957
376	0.00205	0.00221	0.23369	0.21866
386	0.00194	0.00208	0.21377	0.19721
395	0.00184	0.00195	0.19679	0.17909
465	0.00109	0.00105	0.09509	0.07522

**Table 8:** Membership functions and boundaries of fuzzy sets of the input signal “Flying hours”.

	Input Signal	Membership Functions
Flying hours [h]	VERY SMALL [0 0 105] SMALL [0 105 205] MEDIUM [105 205 305] LARGE [205 305 410] VERY LARGE [305 410 500 580]	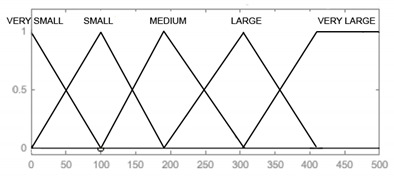

**Table 9:** Membership functions and boundaries of fuzzy sets of the input signal “Number of firing hours”.

	Input Signal	Membership Functions
Firing hours [number of shots]	VERY SMALL [0 0 400] SMALL [0 400 800] MEDIUM [400 800 1300] LARGE [800 1300 1800] VERY LARGE [1300 1800 2000 2000]	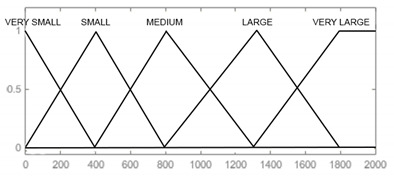

**Table 10:** Membership functions and boundaries of fuzzy sets of the input signal “Corrosion”.

	Input Signal	Membership Functions
Corrosion [mm]	VERY SMALL [0 0 0.02] SMALL [0 0.02 0.04] MEDIUM [0.02 0.04 0.06] LARGE [0.04 0.06 0.075] VERY LARGE [0.06 0.075 0.1 0.1]	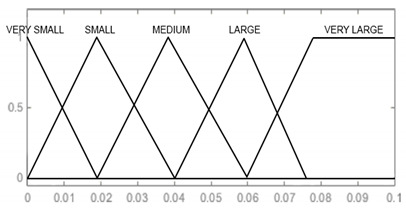

**Table 11:** Membership functions and boundaries of fuzzy sets of the output signal “Reliability”.

	Output Signal	Membership Functions
Reliability [P]	VERY SMALL [0 0 0.2] SMALL [0 0.2 0.4] MEDIUM [0.2 0.4 0.6] LARGE [0.4 0.6 0.8] VERY LARGE [0.6 0.8 1] OPTIMUM [0.8 1 1]	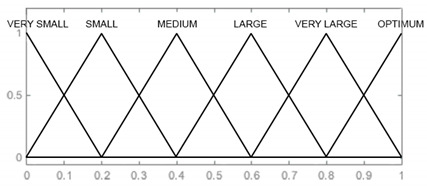

**Table 12:** Values of the reliability function from the fuzzy model.

No	Flying Hours (h)	Shots	Corrosion (mm)	*R*(*t*)
1	65	0	0	0.9300
2	106	0	0	0.9100
3	113	0	0	0.8860
4	133	0	0	0.8430
5	138	0	0	0.8350
6	148	0	0	0.8220
7	156	0	0	0.8130
8	175	0	0	0.7950
9	205	0	0	0.6990
10	214	0	0	0.6650
11	252	0	0	0.5680
12	257	0	0	0.5560
13	268	0	0	0.5310
14	302	0	0	0.4160
15	328	0	0	0.3460
16	376	0	0	0.2470
17	386	0	0	0.2170
18	395	0	0	0.1790
19	465	0	0	0.0633

**Table 13:** Values of the reliability function from the fuzzy model for different defuzzification methods.

No	Flying Hours [h]	Shots	Corrosion [mm]	*R*(*t*) Centre of Gravity Defuzzification Method	*R*(*t*) Method of Defuzzification of the Centre of Sums
1	65	0	0	0.9300	0.9400
2	106	0	0	0.9100	0.9300
3	113	0	0	0.8860	0.9200
4	133	0	0	0.8430	0.8600
5	138	0	0	0.8350	0.8400
6	148	0	0	0.8220	0.8200
7	156	0	0	0.8130	0.8100
8	175	0	0	0.7950	0.7900
9	205	0	0	0.6990	0.7600
10	214	0	0	0.6650	0.7400
11	252	0	0	0.5680	0.5200
12	257	0	0	0.5560	0.5000
13	268	0	0	0.5310	0.4700
14	302	0	0	0.4160	0.4000
15	328	0	0	0.3460	0.3700
16	376	0	0	0.2470	0.2600
17	386	0	0	0.2170	0.1400
18	395	0	0	0.1790	0.1000
19	465	0	0	0.0633	0.0500

**Table 14:** Values of the reliability functions from the classical model (empirical distribution and theoretical distribution) and the “fuzzy” model.

No	Flying Hours (h)	*R*(*t*)—Classical Model (Empirical)	*R*(*t*)—Classical Model (Theoretical)	*R*(*t*)—Fuzzy Model
1	65	0.97315	0.98139	0.9300
2	106	0.92085	0.93809	0.9100
3	113	0.90908	0.92774	0.8860
4	133	0.87118	0.89335	0.8430
5	138	0.86076	0.88365	0.8350
6	148	0.83887	0.86298	0.8220
7	156	0.82040	0.84526	0.8130
8	175	0.77347	0.79919	0.7950
9	205	0.69235	0.71672	0.6990
10	214	0.66680	0.69012	0.6650
11	252	0.55601	0.57214	0.5680
12	257	0.54134	0.55626	0.5560
13	268	0.50923	0.52132	0.5310
14	302	0.41286	0.41546	0.4160
15	328	0.34412	0.33957	0.3460
16	376	0.23369	0.21866	0.2470
17	386	0.21377	0.19721	0.2170
18	395	0.19679	0.17909	0.1790
19	465	0.09509	0.07522	0.0633
